# Cellular sources of dysregulated cytokines in relapsing-remitting multiple sclerosis

**DOI:** 10.1186/1742-2094-9-215

**Published:** 2012-09-14

**Authors:** Jeppe Romme Christensen, Lars Börnsen, Dan Hesse, Martin Krakauer, Per Soelberg Sørensen, Helle Bach Søndergaard, Finn Sellebjerg

**Affiliations:** 1 Department of Neurology, Danish Multiple Sclerosis Center, Copenhagen University Hospital Rigshospitalet, Blegdamsvej 9, Copenhagen, 2100, Denmark

**Keywords:** Relapsing-remitting multiple sclerosis, Immunology, Cytokines, Blood, Cerebrospinal fluid cells, Real-time PCR

## Abstract

**Background:**

Numerous cytokines are implicated in the immunopathogenesis of multiple sclerosis (MS), but studies are often limited to whole blood (WB) or peripheral blood mononuclear cells (PBMCs), thereby omitting important information about the cellular origin of the cytokines. Knowledge about the relation between blood and cerebrospinal fluid (CSF) cell expression of cytokines and the cellular source of CSF cytokines is even more scarce.

**Methods:**

We studied gene expression of a broad panel of cytokines in WB from relapsing-remitting multiple sclerosis (RRMS) patients in remission and healthy controls (HCs). Subsequently we determined the gene expression of the dysregulated cytokines in isolated PBMC subsets (CD4^+^, CD8^+^T-cells, NK-cells, B-cells, monocytes and dendritic cells) from RRMS patients and HCs and in CSF-cells from RRMS patients in clinical relapse and non-inflammatory neurological controls (NIND).

**Results:**

RRMS patients had increased expression of IFN-gamma (*IFNG*), interleukin (IL) 1-beta (*IL1B*), *IL7*, *IL10*, *IL12A*, *IL15*, *IL23*, *IL27*, lymphotoxin-alpha (*LTA*) and lymphotoxin-beta (*LTB*) in WB. In PBMC subsets the main sources of pro-inflammatory cytokines were T- and B-cells, whereas monocytes were the most prominent source of immunoregulatory cytokines. In CSF-cells, RRMS patients had increased expression of *IFNG* and *CD19* and decreased expression of *IL10* and *CD14* compared to NINDs. *CD19* expression correlated with expression of *IFNG, IL7, IL12A, IL15* and *LTA* whereas *CD14* expression correlated with *IL10* expression.

**Conclusions:**

Using a systematic approach, we show that expression of pro-inflammatory cytokines in peripheral blood primarily originates from T- and B-cells, with an important exception of *IFNG* which is most strongly expressed by NK-cells. In CSF-cell studies, B-cells appear to be enriched in RRMS and associated with expression of pro-inflammatory cytokines; contrarily, monocytes are relatively scarce in CSF from RRMS patients and are associated with IL10 expression. Thus, our findings suggest a pathogenetic role of B-cells and an immunoregulatory role of monocytes in RRMS.

## Introduction

Multiple sclerosis (MS) is a chronic, immune-mediated disease of the central nervous system (CNS). An interplay among genetic susceptibility and environmental factors is implicated in the pathogenesis of MS. In relapsing-remitting MS (RRMS), disability develops with relapses. The pathological correlates of clinical relapses are focal, transient attacks of immune cells on myelin and axons, resulting in the formation of an MS plaque [1]. In pathology studies of brain tissue from RRMS patients, T- and B-cells are seen accumulating in perivascular cuffs near MS plaques and to a lesser extent in the parenchyma, while monocytes and macrophages are seen mostly in the lesion parenchyma. In the cerebrospinal fluid (CSF) many MS patients show a mild pleocytosis. T-cells are the major cell subset in CSF, and compared to non-inflammatory neurological disease (NIND) an increase in B-cells and plasma cells and a decrease in monocytes and natural killer (NK) cells is observed in MS patients [2]. The relation between the peripheral immune system and CNS inflammation in RRMS is underscored by the efficacy of treatment with the monoclonal antibodies natalizumab [3] and rituximab [4], which exert their effect on peripheral immune cells, but have a major impact on disease activity, cell numbers, levels of cytokines and markers of tissue damage in CSF [5-7]. During immune attacks on myelin, peripheral activation and subsequent migration of autoreactive T-helper type 1 (Th1) and Th17 cells to the CNS is an essential step [8]. How the T-cells become activated remains unclear, but antigen-presenting cells (APCs) located in peripheral lymph nodes [9] and the subarachnoidal space [10] are likely to be involved.

Gene expression studies of brain tissue, peripheral blood mononuclear cells (PBMCs) and whole blood (WB) from MS patients have shown dysregulated expression of cytokines, chemokines, and transcription factors related to immune activation [11]. Interferon (IFN)-gamma, the Th1 signature cytokine, has been implicated in the pathogenesis of MS, and induced disease activity in a clinical trial [12], although in the animal model experimental autoimmune encephalomyelitis (EAE), *Ifng* knockout mice developed severe disease [13]. In addition to Th1-cells, Th17-cells that secrete interleukin (IL)-17 are believed to be important in the pathogenesis [8].

Since gene expression studies of molecules used in biomarker studies are mostly carried out on WB or PBMCs, the cellular origin of cytokines is usually not determined. To date, no study has systematically analyzed the cellular origin of dysregulated cytokines in MS patients. Furthermore little is known about cytokine expression in CSF-cells and the cellular source of CSF cytokines. CSF studies are sparse due to the limited availability of CSF, but studies linking peripheral and CSF immune responses are central to understanding the immunopathogenesis of MS, and to translate the many findings in studies of peripheral immune activation.

In the present study, we used gene expression analysis to identify dysregulated cytokines in WB from RRMS patients in clinical remission and subsequently investigated the cellular source of these cytokines in isolated PBMC subsets. Furthermore, to relate the findings in peripheral blood to CNS inflammation, we studied gene expression of the dysregulated cytokines in CSF-cells from RRMS patients in relapse, and determined whether it correlated with markers for T-, B- and NK-cells and monocytes in the CSF.

## Materials and methods

### Subjects

An initial cohort of 39 untreated RRMS patients in clinical remission and 39 healthy controls (HCs) was recruited for studies of gene expression in WB (Table 1). A second cohort of four untreated RRMS patients in remission and four HCs was recruited for studies of gene expression in isolated PBMC subsets. Finally a third cohort of 17 untreated RRMS patients in relapse and 10 NIND patients (3 with spinal stenosis, 3 with herniated lumbar discs and 4 with low back pain) were recruited for studies on CSF-cells and PBMCs; CSF sampling was done prior to initiation of eventual steroid pulse therapy. All RRMS cohorts studied consisted of both recent onset RRMS and RRMS with longer disease duration (Table 1). The studies were approved by the local scientific ethics committee and informed consent was obtained from all patients and healthy controls.

**Table 1:** Demographic and clinical characteristics of the cohorts

	**RRMS patients**				**Control groups**	
	**Median age**	**Female%**	**Median EDSS**	**Duration**	**Mean age**	**Female%**
**Whole blood studies**	34.0 (28.0 to 39.0)	59%	2.0 (1.0 to 3.0)	5.0 (2.0 to 7.0)	32.0 (29.0 to 38.0)	58%
**Cell subset studies**	38.0 (30.5 to 47.0)	75%	1.5 (1.0 to 3.1)	5.0 (1.3 to 12.5)	33.5 (24.5 to 36.5)	75%
**CSF cell studies**	37.5 (31.3 to 42.8)	59%	3.5 (3.0 to 6.4)	6.0 (1.5 to 9.75)	51.5 (45.0 to 59.0)	70%

Median values with interquartile ranges in brackets. EDSS, Expanded Disability Status Scale; RRMS, relapsing-remitting multiple sclerosis.

### Gene expression studies

WB was sampled in PAXgene tubes and RNA extracted using PAXgene Blood RNA Kit (PreAnalytiX, Hombrechtikon, Switzerland); cDNA synthesis was done with High Capacity cDNA Reverse Transcription Kit (Applied Biosystems, Foster City, California, USA). For studies of gene expression in PBMC subsets, PBMCs were isolated using Lymphoprep (Axis-Shield, Oslo, Norway) and subsets of CD4^+^T-cells, CD8^+^T-cells, NK-cells, B-cells, monocytes and dendritic cells (DC) were isolated using MACS cell separation kits (CD4^+^T Cell Isolation Kit, CD8^+^T Cell Isolation Kit, NK Cell Isolation Kit, CD19 MicroBeads, CD14 MicroBeads and Blood Dendritic Cell Isolation Kit) and an autoMACS separator (all from Miltenyi Biotec, Bergisch Gladbach, Germany). Mean purities of the PBMC subsets were above 93%, except for NK-cells which had a mean purity of 73%. RNA was extracted from 80,000 to 200,000 cells of the obtained subsets with PicoPure RNA Isolation Kit (Arcturus, Mountain View, California, USA). cDNA synthesis was done with qScript cDNA SuperMix (Quanta BioSciences, Gaithersburg, Maryland, USA). For CSF-cell gene expression studies, CSF was obtained by lumbar puncture within 30 days from the onset of a relapse with no evidence of spontaneous recovery. Blood was sampled on the same day as the lumbar puncture and PBMCs were isolated with Lymphoprep. We snap froze at least 5,000 CSF-cells and 200,000 PBMCs for later RNA extraction with PicoPure RNA Isolation Kit and cDNA synthesis with High Capacity cDNA Reverse Transcription Kit.

In the selection of the genes of interest (Table 2), we focused on genes that have been shown to be involved in the pathogenesis of MS, genes involved in the expression of Th1 and Th17 cytokines, and genes expressed in APCs. In a pilot study in the WB cohort we excluded *IL1A*, *IL2*, *IL4*, *IL5*, *IL6*, *IL9*, *IL12B*, *IL13*, *IL17A*, *IL17F*, *IL21*, *IL33*, *GM-CSF* and *BDNF* from analysis as candidate biomarkers due to low expression levels in WB samples. Real-time polymerase chain reactions (RT-PCR) were performed with TaqMan Gene Expression Assays on a 7500 Real-Time PCR System (Applied Biosystems, USA). Threshold cycle (CT) values were calculated using SDS software (Applied Biosystems, USA). In CSF-cell samples without amplification the expression value was arbitrarily set to 0. The relative mRNA transcript expression was calculated by the comparative CT method also referred to as the 2^-ΔΔCT^ method, using GAPDH as reference gene. Expression values were normalized to a HC PBMC cDNA pool resulting in a normalization ratio (NR):

(1) $$NR=2^{-\Delta \Delta CT},\text{where}\;\Delta \Delta CT=\left(CT_{target/sample}-CT_{target/pool}\right)-\left(CT_{GAPDH/sample}-CT_{GAPDH/pool}\right)$$

**Table 2:** List of TaqMan Gene Expression Assays used in the gene expression studies

**Gene Symbol**	**Assay number**	**GenBank**	**Whole blood**	**PBMC populations**	**CSF cells**
***GAPDH***	Hs99999905_m1	NM_002046	·	·	·
***IFNG***	Hs99999041_m1	NM_000619	·	·	·
***IL1B***	Hs00174097_m1	NM_000576	·	·	·
***IL1RN***	Hs00277299_m1	NM_000577	·		
***IL7***	Hs00174202_m1	NM_000880	·	·	·
***IL10***	Hs00174086_m1	NM_000572		·	·
***IL12A***	Hs00168405_m1	NM_000882	·	·	·
***IL15***	Hs00542571_m1	NM_172175	·	·	·
***IL18***	Hs00155517_m1	NM_001562	·		
***IL23***	Hs00372324_m1	NM_016584		·	·
***IL27***	Hs00377366_m1	NM_145659	·	·	·
***EBI3***	Hs00194957_m1	NM_005755	·		
***LTA***	Hs00236874_m1	NM_000595	·	·	·
***LTB***	Hs00242739_m1	NM_009588	·	·	·
***TGFB1***	Hs99999918_m1	NM_000660	·		
***TNF***	Hs00174128_m1	NM_000594	·		
***PRF1***	Hs00169473_m1	NM_005041	·		
***CD3d***	Hs00174158_m1	NM_000732		·	·
***CD14***	Hs00169122_g1	NM_000591		·	·
***CD19***	Hs01047407_m1	NM_001770		·	·
***CD56***	Hs00941824_m1	NM_181351		·	·

Assay numbers listed represents TaqMan Gene Expression Assays used. CSF, cerebrospinal fluid; GenBank, GenBank accession numbers; PBMC, peripheral blood mononuclear cell.

### Statistical analysis

Statistical analysis was performed using PASW 18 software (IBM, Armonk, New York, USA). For comparison between independent groups, non-parametric Mann–Whitney tests were used. Wilcoxon signed rank tests were used for analysis of paired samples. For correlation analysis, we used Spearman's rank correlation coefficient. Changes in the expression of mRNA were considered significant for *P* <0.05. Since the analysis of gene expression data involved multiple comparisons, we also used the false discovery rate (FDR) method to calculate q-values [14]. Q-values were considered significant for q <0.05.

## Results

### Gene expression in whole blood from RRMS patients in remission

**Figure 1:**
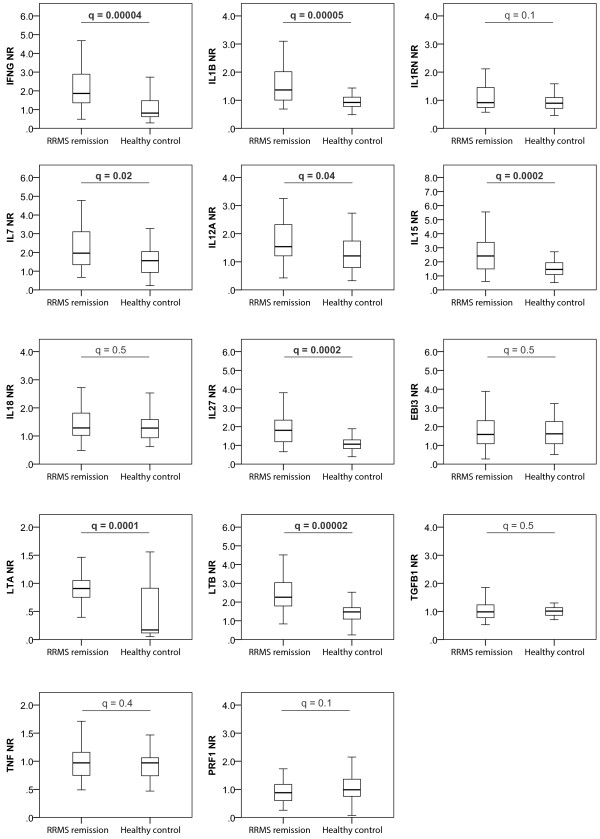
**Cytokine gene expression in whole blood from multiple sclerosis patients and healthy controls.** Whole blood gene expression data from relapsing-remitting multiple sclerosis (RRMS) patients in remission and healthy controls are shown as the normalization ratio (NR). Mann–Whitney tests were used for testing differences between groups, and q-values represent false discovery rate corrected *P*-values. Significant test-values are in bold.

Initially, we compared the WB expression of cytokine genes in RRMS patients in remission and HCs. We found significantly increased expression of IFN-gamma (*IFNG*), *IL1B, IL7IL12AIL15IL27*, lymphotoxin-alpha *(LTA*) and lymphotoxin-beta (*LTB*) in RRMS (Figure 1). In previous studies conducted in the same cohort we found increased expression of *IL10* and *IL23*[15]. 

### Gene expression in PBMC cell subpopulations from RRMS patients in remission

**Figure 2:**
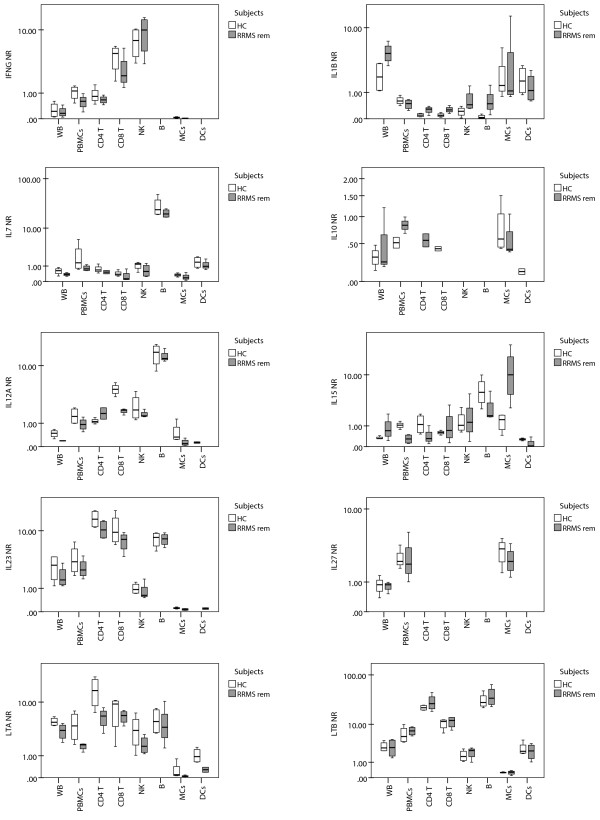
**Sources of cytokines in peripheral blood cell subsets.** Box-plots of cytokine gene expression for different cell populations are isolated from relapsing-remitting patients in remission (RRMS rem) and healthy controls (HC). Gene expression data are shown as the normalization ratio (NR).

To substantiate the findings of increased cytokine gene expression in WB from RRMS patients in remission, we conducted gene expression studies on PBMC subsets from four RRMS patients in remission and four HCs. Due to the low number of subjects, we did not expect to find differences between the two groups. As shown in Figure 2, the cellular sources of *IFNG* are mainly NK-cells, CD8^+^T-cells and, to a lesser extent, CD4^+^T-cells. This is not unexpected, but highlights NK-cells as a major source of *IFNG* and thus questions the use of *IFNG* as a specific Th1 biomarker in peripheral blood studies without detailing the cellular source. For *IL1B,* the cellular sources are mainly monocytes and DCs. Notably, *IL1B* expression in WB is higher than in PBMCs, reflecting that granulocytes are a major source of *IL1B* in WB. *IL10* is expressed in monocytes, CD4^+^ and CD8^+^T-cells. *IL7* and *IL12A* are expressed mainly in B-cells and *IL15* mainly in B-cells and monocytes. For *IL23,* the cellular sources are CD4^+^T-cells, CD8^+^T-cells and B-cells. Unexpectedly, we observed low expression in monocytes and DCs. This finding contradicts the current understanding of IL23, which is thought to be an APC cytokine. However, since *IL23A* transcripts are translated into IL23p19 protein, which together with IL12p40 form bioactive IL23, and IL12p40 production is known to be limited to activated monocytes and DCs [16] and B-cells [17], the biological role of *IL23* expression in T-cells is unclear. Thus, our findings indicate that *in vivo* WB *IL23* gene expression is not suitable for studying IL23, which should instead be studied at the protein level as IL-23 p19/IL-12 p40 heterodimer. *IL27* expression is restricted to monocytes. *LTA* is mainly expressed in CD4^+^T-cells, CD8^+^T-cells and B-cells and, to a lesser extent, in NK-cells while *LTB* is mainly expressed in B-cells, CD4^+^T-cells and at lower levels in CD8^+^T-cells.

### Gene expression in PBMCs and CSF-cells from RRMS relapse patients

Next, we wanted to relate the findings of dysregulated cytokines in peripheral blood cells with CNS inflammation. We analyzed the cytokine expression in CSF-cells and PBMCs from a cohort of RRMS relapsed and NIND patients (Table 3). *IL10* expression was significantly enriched in CSF-cells compared to PBMCs with a 9-fold increase in RRMS relapse patients and a 42-fold increase in NINDs. *LTB* expression was higher in CSF-cells than in PBMCs with a 2.4-fold increase in RRMS relapsed patients and a 1.7-fold increase in NINDs. *IL1B* expression in CSF-cells was 0.18-fold lower than in PBMCs in RRMS relapsed patients. Compared with NIND, RRMS patients had increased *IFNG* and decreased *IL10* expression in CSF-cells. The *IFNG* and *LTA* CSF/PBMC ratios were significantly higher in RRMS relapsed patients, while the *IL10* CSF/PBMC ratio was lower in RRMS than in NIND patients. In CSF-cells *IL7* and *IL15* expression was only detectable in a few samples (all from RRMS patients).

**Table 3:** Cytokine expression in cerebrospinal fluid cells and peripheral blood mononuclear cells

		**RRMS relapse**			**NIND**			**RRMS relapse vs NIND**	
	**Mean CT value**	**N**	**Median (IQR)**	**CSF/PBMC p-value**	**N**	**Median (IQR)**	**CSF/PBMC p-value**	**p-value**	**q-value**
***IFNG*PBMC NR**	32.3	15	4.10 (2.74 to 10.21)		10	5.25 (2.65 to 9.12)		0.78	0.37
***IFNG*CSF NR**	34.9	15	7.22 (0.00 to 16.42)		10	0.00 (0.00 to 0.00)		**0.004**	**0.02**
***IFNG*CSF/PBMC Ratio**		15	0.71 (0.00 to 2.31)	0.87	10	0.00 (0.00 to 0.00)		**0.004**	**0.02**
***IL1B*PBMC NR**	32.4	16	1.13 (0.88 to 2.14)		10	1.81 (1.05 to 2.70)		0.19	0.15
***IL1B*CSF NR**	36.7	14	0.21 (0.00 to 1.09)		10	2.70 (0.00 to 6.94)		0.36	0.23
***IL1B*CSF/PBMC Ratio**		13	0.18 (0.00 to 0.92)	**0.02**	10	1.96 (0.00 to 6.40)	0.29	0.42	0.24
***IL7*PBMC NR**	34.0	16	0.41 (0.21 to 1.17)		10	0.31 (0.18 to 0.46)		0.40	0.24
***IL7*CSF NR**	36.1	13	0.00 (0.00 to 0.86)		10	0.00 (0.00 to 0.00)		0.06	0.09
***IL7*CSF/PBMC Ratio**		12	0.00 (0.00 to 2.51)		8	0.00 (0.00-0.00)		0.08	0.09
***IL10*PBMC NR**	34.3	16	2.75 (2.02 to 3.90)		10	2.95 (2.38 to 4.26)		0.54	0.27
***IL10*CSF NR**	35.3	17	23.51 (3.22 to 46.39)		10	116.78 (72.22 to 156.28)		**0.001**	**0.01**
***IL10*CSF/PBMC Ratio**		16	8.57 (0.79 to 12.71)	**0.003**	10	42.21 (24.04 to 66.18)	**0.01**	**0.002**	**0.01**
***IL12A*PBMC NR**	37.5	16	0.49 (0.30 to 0.66)		10	0.54 (0.42 to 0.82)		0.53	0.27
***IL12A*CSF NR**	38.3	15	0.00 (0.00 to 0.00)		10	0.00 (0.00 to 0.00)		0.24	0.17
***IL12A*CSF/PBMC Ratio**		12	0.00 (0.00 to 0.00)		9	0.00 (0.00 to 0.00)		0.21	0.16
***IL15*PBMC NR**	36.3	16	1.19 (0.91 to 1.43)		10	1.10 (0.57 to 1.60)		0.79	0.37
***IL15*CSF NR**	37.3	14	0.00 (0.00 to 1.10)		10	0.00 (0.00 to 0.00)		0.07	0.09
***IL15*CSF/PBMC Ratio**		13	0.00 (0.00 to 0.97)		10	0.00 (0.00 to 0.00)		0.06	0.09
***IL23*PBMC NR**	34.6	16	0.80 (0.65 to 1.18)		10	0.80 (0.62 to 1.15)		1.00	0.44
***IL23*CSF NR**	36.8	14	1.12 (0.00 to 1.98)		10	0.00 (0.00 to 0.48)		0.10	0.10
***IL23*CSF/PBMC Ratio**		13	1.54 (0.00 to 3.17)	0.28	10	0.00 (0.00 to 1.50)		0.09	0.10
***IL27*PBMC NR**	35.1	16	4.74 (2.49 to 7.60)		10	8.36 (6.17 to 12.70)		**0.045**	0.09
***IL27*CSF NR**	ND	14	0.00 (0.00 to 0.00)		10	0.00 (0.00 to 0.00)		1.00	0.44
***IL27*CSF/PBMC Ratio**		12	0.00 (0.00 to 0.00)		10	0.00 (0.00 to 0.00)		1.00	0.44
***LTA*PBMC NR**	34.3	16	1.74 (1.43 to 2.30)		10	2.11 (1.59 to 2.73)		0.37	0.23
***LTA*CSF NR**	36.7	14	1.41 (0.00 to 3.20)		10	0.00 (0.00 to 0.00)		0.05	0.09
***LTA*CSF/PBMC Ratio**		13	1.08 (0.00 to 1.82)	0.92	10	0.00 (0.00 to 0.00)		**0.04**	0.09
***LTB*PBMC NR**	28.2	16	4.09 (2.44 to 4.99)		10	3.71 (3.06 to 5.54)		0.79	0.37
***LTB*CSF NR**	33.5	13	8.05 (6.62 to 13.37)		10	8.82 (3.30 to 12.27)		0.42	0.23
***LTB*CSF/PBMC Ratio**		12	2.37 (1.76 to 3.12)	**0.002**	10	1.70 (0.97 to 3.12)	**0.04**	0.13	0.11

Gene expression data are shown as the normalization ratio (NR). Mean cycle threshold (CT) is shown for each target. CSF/PBMC ratio represent fold change in expression between CSF cells and PBMCs. CSF/PBMC *P*-values represents Wilcoxon signed rank test results, and tests were only conducted if median CSF NR was >0. All other *P*-values represent Mann–Whitney tests for differences between groups, and q-values represent false discovery rate corrected *P*-values. Significant test-values are in bold. CSF, cerebrospinal fluid; IQR, Interquartile range; NIND, non-inflammatory neurological disease; PBMC; peripheral blood mononuclear cell; RRMS relapse, relapsing-remitting multiple sclerosis patients in relapse.

### CSF-cell subsets and cytokine gene expression in RRMS relapse patients

To elucidate whether differences in cytokine gene expression are associated with the cell subsets present in CSF, we analysed gene expression of *CD3d*, *CD14*, *CD19* and *CD56* (markers of T-cells, monocytes, B-cells and NK-cells and NK T-cells, respectively) in CSF-cells (Table 4). Comparing expression of cell type markers in CSF-cells to PBMCs, we found significantly increased expression of *CD3d* in CSF-cells in RRMS relapse patients and increased *CD14* expression in CSF-cells from NINDs. RRMS relapse patients had a decrease in the expression of *CD14* and the *CD14* CSF/PBMC ratio and an increase in expression of *CD19* and the *CD19* CSF/PBMC ratio compared with NIND patients.

**Table 4:** Expression of markers of cell types in cerebrospinal fluid cells and peripheral blood mononuclear cells

		**RRMS relapse**			**NIND**			**RRMS relapse vs NIND**	
	**Mean CT**	**N**	**Median (IQR)**	**CSF/PBMC p-value**	**N**	**Median (IQR)**	**CSF/PBMC p-value**	**p-value**	**q-value**
***CD3d*PBMC NR**	31.1	15	0.06 (0.04 to 0.08)		10	0.05 (0.04 to 0.07)		0.47	0.26
***CD3d*CSF NR**	35.6	13	0.10 (0.08 to 0.23)		10	0.06 (0.02 to 0.15)		0.07	0.09
***CD3d*CSF/PBMC Ratio**		11	2.37 (1.30 to 3.67)	**0.01**	10	1.12 (0.42 to 3.07)	0.39	0.23	0.17
***CD14*PBMC NR**	30.1	15	0.33 (0.27 to 0.38)		10	0.33 (0.29 to 0.49)		0.38	0.23
***CD14*CSF NR**	34.9	13	0.38 (0.07 to 0.52)		10	1.52 (0.94 to 2.40)		**0.0004**	**0.01**
***CD14*CSF/PBMC Ratio**		11	1.39 (0.30 to 2.15)	0.42	10	4.01 (3.54 to 5.16)	**0.01**	**0.003**	**0.02**
***CD19*PBMC NR**	36.1	15	0.14 (0.09 to 0.23)		10	0.08 (0.05 to 0.16)		0.05	0.09
***CD19*CSF NR**	37.1	13	0.00 (0.00 to 0.35)		10	0.00 (0.00 to 0.00)		**0.03**	0.09
***CD19*CSF/PBMC Ratio**		11	0.00 (0.00 to 0.89)		10	0.00 (0.00 to 0.00)		**0.04**	0.09
***CD56*PBMC NR**	34.9	15	0.15 (0.09 to 0.23)		10	0.16 (0.07 to 0.32)		0.96	0.43
***CD56*CSF NR**	37.8	13	0.00 (0.00 to 0.03)		10	0.00 (0.00 to 0.00)		0.11	0.10
***CD56*CSF/PBMC Ratio**		11	0.00 (0.00 to 0.00)		10	0.00 (0.00 to 0.00)		0.17	0.14

Gene expression data are shown as normalization ratio (NR). Mean cycle threshold (CT) is shown for each target. CSF/PBMC ratio represent fold change in expression between CSF cells and PBMCs. CSF/PBMC p-values represents Wilcoxon signed rank test results, and tests were only conducted if median CSF NR was >0. All other p-values represent Mann–Whitney tests for difference between groups, and q-values represent false discovery rate corrected p-values. Significant test-values are in bold. RRMS relapse: Relapsing-remitting multiple sclerosis patients in relapse. NIND: Non-inflammatory neurological disease. PBMC: Peripheral blood mononuclear cell. CSF: Cerebrospinal fluid. IQR: Interquartile range.

Correlations between CSF cytokine expression and cell type markers were analyzed using Spearman’s rank correlation analysis. Since several of the gene targets were only expressed in some patients, resulting in low statistical power, we analyzed correlations for the whole cohort and for the cohort of RRMS patients in relapse (Table 5). For the whole cohort, *INFG* and *CD19* expression correlated positively, and *INFG* and *CD14* expression correlated negatively. *IL10* expression correlated positively with *CD14* and negatively with *CD19*. *IL10* expression in CSF-cells correlated significantly with *CD14* expression, suggesting that monocytes are an important source of *IL10* in CSF-cells*.* Consistent with the PBMC subset findings, we further found positive correlations between *CD19* and *IFNG, IL7, IL12A, IL15* and *LTA; CD3d* and *LTB; CD56* and *IL12A.* These findings also reached statistical significance when FDR-corrected q-values were calculated. For RRMS relapse patients some of the correlations were confirmed, but none of them reached statistical significance when q-values were calculated.

**Table 5:** Correlations between cytokine and cell type marker expression in cerebrospinal fluid cells

			**RRMS relapse and NIND**				**RRMS relapse**			
**CSF NR**			**N**	**R**	**p-value**	**q-value**	**N**	**R**	**p-value**	**q-value**
***IFNG***	vs	***CD3d***	22	0.383	0.08	0.06	12	0.441	0.151	0.39
***IFNG***	vs	***CD14***	22	−0.606	**0.003**	**0.008**	12	0.018	0.956	0.90
***IFNG***	vs	***CD19***	22	0.538	**0.010**	**0.02**	12	0.111	0.731	1.04
***IFNG***	vs	***CD56***	22	0.372	0.09	0.06	12	0.019	0.954	0.93
***IL1B***	vs	***CD3d***	23	−0.453	**0.03**	**0.03**	13	0.064	0.837	0.89
***IL1B***	vs	***CD14***	23	0.126	0.57	0.27	13	0.067	0.829	0.91
***IL1B***	vs	***CD19***	23	0.168	0.44	0.21	13	0.435	0.137	0.39
***IL1B***	vs	***CD56***	23	0.110	0.62	0.28	13	0.285	0.345	0.73
***IL7***	vs	***CD3d***	23	0.090	0.68	0.30	13	−0.121	0.694	1.03
***IL7***	vs	***CD14***	23	−0.169	0.44	0.22	13	0.087	0.777	0.98
***IL7***	vs	***CD19***	23	0.600	**0.002**	**0.009**	13	0.513	0.073	0.36
***IL7***	vs	***CD56***	23	0.412	0.05	**0.04**	13	0.241	0.429	0.86
***IL10***	vs	***CD3d***	23	−0.506	**0.01**	**0.02**	13	**−0.589**	**0.034**	0.23
***IL10***	vs	***CD14***	23	0.773	**0.00002**	**0.00011**	13	0.541	0.056	0.32
***IL10***	vs	***CD19***	23	−0.438	**0.04**	**0.04**	13	−0.151	0.623	1.01
***IL10***	vs	***CD56***	23	−0.242	0.27	0.15	13	0.086	0.781	0.95
***IL12A***	vs	***CD3d***	23	0.075	0.73	0.31	13	−0.096	0.755	1.03
***IL12A***	vs	***CD14***	23	−0.327	0.13	0.08	13	−0.236	0.437	0.78
***IL12A***	vs	***CD19***	23	**0.577**	**0.004**	**0.008**	13	0.508	0.076	0.32
***IL12A***	vs	***CD56***	23	**0.756**	**0.00003**	**0.00014**	13	0.697	**0.008**	0.09
***IL15***	vs	***CD3d***	23	0.226	0.30	0.16	13	0.067	0.828	0.94
***IL15***	vs	***CD14***	23	**−0.519**	**0.01**	**0.02**	13	−0.450	0.123	0.42
***IL15***	vs	***CD19***	23	**0.847**	**0.0000003**	**0.0000048**	13	0.758	**0.003**	0.09
***IL15***	vs	***CD56***	23	**0.469**	**0.02**	**0.03**	13	0.349	0.242	0.59
***IL23***	vs	***CD3d***	23	0.004	0.98	0.39	13	0.034	0.912	0.91
***IL23***	vs	***CD14***	23	−0.396	0.06	0.05	13	−0.040	0.898	0.92
***IL23***	vs	***CD19***	23	0.340	0.11	0.07	13	0.177	0.562	0.96
***IL23***	vs	***CD56***	23	0.234	0.28	0.15	13	0.092	0.766	1.00
***LTA***	vs	***CD3d***	23	0.002	0.99	0.39	13	−0.121	0.693	1.07
***LTA***	vs	***CD14***	23	−0.420	0.05	0.04	13	−0.078	0.800	0.94
***LTA***	vs	***CD19***	23	**0.563**	**0.005**	**0.009**	13	0.461	0.113	0.43
***LTA***	vs	***CD56***	23	0.384	0.07	0.05	13	0.238	0.433	0.82
***LTB***	vs	***CD3d***	23	**0.579**	**0.004**	**0.009**	13	0.440	0.133	0.41
***LTB***	vs	***CD14***	23	−0.057	0.80	0.33	13	0.305	0.310	0.70
***LTB***	vs	***CD19***	23	−0.337	0.12	0.07	13	−0.702	**0.008**	0.13
***LTB***	vs	***CD56***	23	−0.304	0.16	0.09	13	−0.598	**0.031**	0.26

Gene expression data are shown as the normalization ratio (NR). Correlation analysis was done with Spearman’s rank correlation coefficient, and q-values represent the false discovery rate corrected *P*-values. Significant values are in bold. RRMS relapse: Relapsing-remitting multiple sclerosis patients in relapse. CSF cerebrospinal fluid; NIND, Non-inflammatory neurological disease; PBMC, Peripheral blood mononuclear cell.

## Discussion

In the present study we find an increased expression of pro-inflammatory (*INFG*, *IL1B*, *IL7*, *IL12A*, *IL15*, *IL23*, *LTA* and *LTB*) and immunoregulatory cytokines (*IL10* and *IL27*) in WB from RRMS patients in remission. In subsequent gene expression studies on PBMC subsets, our findings confirm the expected sources for many cytokines, but for others we reveal unexpected cell-types to be major sources. In general, T- and B-cells are the most frequent sources of the pro-inflammatory cytokines, and monocytes are the predominant source of the immunoregulatory cytokines. In CSF-cell studies, we demonstrate that the majority of the cytokines are expressed by CSF-cells, and that CSF-cells from RRMS patients have increased expression of *IFNG* and markers of T- and B-cells, whereas *IL10* and the marker for monocytes have decreased expression. Finally, the B-cell marker *CD19* correlated positively with many pro-inflammatory cytokines in CSF-cells.

We present a systematic approach to determine the cellular source of dysregulated cytokines in RRMS, which is critical in the interpretation of findings in cytokine biology. Our studies addressed the expression of genes that may serve as biomarkers of fundamental pathogenic processes in MS in fresh blood samples, which allows studying cytokine biology close to *in vivo* conditions, without the need for *in vitro* stimulation. However, *in vivo* studies have limitations, since they do not address the full potential for activation of the cells in the same way that *in vitro* studies do. Furthermore, gene expression does not necessarily result in translation into bioactive protein, and post-translational modifications or regulated secretion are not reflected by mRNA expression studies. The studies on isolated cell-subsets were generally performed on samples with high purity, but for NK-cells the purity was 73% on average. This could represent a potential bias, as genes not normally detected in NK-cells possibly will be detected. Thus, for genes with low expression levels in NK-cells, conclusions must be very cautious. On the other hand, the very high expression of *IFNG* by NK-cells is not likely to be a result of this impurity. The CSF-cell study had limited statistical power due to the relatively low number of subjects and the low amount of mRNA that could be extracted from these cells. This implies that more target molecules might have been detected if more mRNA had been available. Caution is therefore important in the interpretation of differences in expression levels between PBMCs and CSF-cells in MS patients and controls. Not only will differences in the cellular composition of CSF samples in MS patients and neurological controls confound the results obtained, but differences in the composition of blood and CSF-cell populations common to patients and controls should also be taken into account. Adding to this, specific cell-subsets might change their phenotype or gene expression patterns upon crossing the blood–brain-barrier [18,19].

Looking into the possible roles of specific cytokines found to be dysregulated in our patients with RRMS, IFN-gamma is known to be present in brain lesions in MS patients [20], and IFN-gamma secretion is increased in T-cells from RRMS patients in the blood and even more in CSF. Experimental studies indicate that IFN-gamma, at least when expressed by CD4^+^T-cells, has important effects along with IL17 in the development of RRMS [8]. Our findings of increased *IFNG* in WB and CSF-cells in RRMS patients are in accordance with previous studies, but the finding of NK-cells and CD8^+^T-cells as the major sources highlights that *IFNG* expression in WB is associated with other cellular responses than Th1. As NK-cells have been attributed an immunoregulatory role in MS, this could explain the known ambiguous effects of IFN-gamma in MS and animal models of MS, and our finding emphasizes that further clarification of the function of IFN-gamma in MS requires studies of NK-cells and CD8^+^T-cells. The finding of increased *IFNG* expression in CSF-cells is more likely attributed a pro-inflammatory role in T-cells, and interestingly correlates with B-cells.

We have previously demonstrated negative correlation between expression of the immunoregulatory cytokine *IL10* and the number of active magnetic resonance imaging lesions [21], and IL10 has been suggested to have beneficial effects in MS in numerous studies [22,23]. IL10 is present in perivascular macrophages in MS lesions [24] and is increased in CSF from MS patients [25]. In blood, studies of IL10 expression have been conflicting [25-27] but most biomarker studies point to a slight increase in *IL10* gene expression in MS. Accordingly, we find IL10 expression to be increased in WB from RRMS in remission compared to HCs. In CSF-cells we find a decrease in *IL10* mRNA expression in RRMS relapse compared to NIND patients. This corresponds to a study showing that IL10-protein is decreased during relapses [28], whereas data from a study on a mixed MS group and a non-inflammatory control group [29], reported increased *IL10* mRNA expression in in CSF-cells from MS. The latter study used a mixed MS group where the majority was not in relapse, and hence a likely explanation for the conflicting results is the differences in the specific patient groups studied. Finally, since *IL10* expression was pronounced in monocytes in our PBMC subset study and correlated negatively with *CD3d* and *CD19* and positively with *CD14* in CSF-cells, our findings support that monocytes may have an immunoregulatory role in MS. However, the decreased CSF-cell *IL10* expression in RRMS compared to NIND patients could also simply reflect a relatively decreased frequency of monocytes in MS patients [2] or a change in the monocyte phenotype, since monocytes transmigrating across the blood–brain barrier change their phenotype [19], and CSF monocytes have decreased *CD14* expression [18,30].

IL7 and IL15 are IL-2 family cytokines regulating survival and activation of lymphocytes, and are of interest in MS research since they signal through the IL2- and IL7-receptors, which have shown strong association with MS in genome-wide association studies [31]. Expression of these cytokines is increased in MS brain lesions [32,33], in CSF [34,35] and in blood from MS patients [36,37]. The finding of increased WB expression is in accordance with previous studies on blood cells. Since IL7 and IL15 both contribute to increased T-cell survival and Th1 induction, our findings could represent a B-cell and monocyte-driven pro-inflammatory response in the peripheral immune compartment of MS patients. On the other hand IL15 is also essential for NK-cell survival and IL15-deficiency in mice results in worsening of EAE [38], why caution in the interpretation of the *IL15* findings should be stressed.

The finding of *IL23* expression being most pronounced in T-and B-cells, make clear that a systematic approach can be necessary to derive proper conclusions about the biology behind cytokine gene expression, and in this particular example, that the biological significance of *in vivo IL23* expression is unclear and will demand studies of *IL23* expression in T-cells.

Lymphotoxin is present in MS lesions [39] and expression is increased in blood and CSF in MS [40,41]. Being important for the crosstalk between APCs and T-cells and for the development of ectopic follicle-like structures seen in MS and other autoimmune diseases [42,43] our finding of increased lymphotoxin in WB and CSF-cells points to a probable pro-inflammatory function of this cytokine in MS, although studies proving that the increased mRNA expression is associated with increased bioactivity are needed to substantiate this hypothesis.

## Conclusion

In conclusion, we have confirmed previous findings of dysregulated cytokines in WB in MS patients, particularly increased expression of pro-inflammatory cytokines. A detailed analysis of the cytokine expression in PBMC subsets confirmed the expected origin for some cytokines, while other cytokines also were expressed in unexpected subsets. Most pro-inflammatory cytokines were expressed by B-cells in blood and correlated with *CD19* in CSF-cells. Monocytes were the predominant source of immunoregulatory cytokines, and **IL10** was decreased in CSF-cells compared to NINDs, and correlated with *CD14* in CSF-cells. These findings correspond to studies on CSF-cells in MS [44,45] and a study showing that the most variable cell parameter in CSF is the B-cell/monocyte ratio, which also correlates with disease progression [2]. Thus our study supports a central role of B-cells in the pathogenesis of MS. Recently this has been highlighted by studies proving the benefit of treatment with B cell-depleting antibodies in MS [46].

## Abbreviations

APC: Antigen-presenting cell; CNS: Central nervous system; CSF: Cerebrospinal fluid; CT: Threshold cycle; DC: Dendritic cell; EAE: Experimental autoimmune encephalomyelitis; EDSS: Expanded Disability Status Scale; FDR: False discovery rate; HC: Healthy controls; IFN: Interferon; IFNG: Interferon-gamma; IL: Interleukin; LTA: Lymphotoxin-alpha; LTB: Lymphotoxin-beta; MS: Multiple sclerosis; NIND: Non-Inflammatory neurological disease; NK-cell: Natural killer cell; NR: Normalization ratio; PBMC: Peripheral blood mononuclear cell; RRMS: Relapsing-remitting MS; RT-PCR: Real-time polymerase chain reactions; Th1: T-helper type 1; Th17: T-helper type 17; WB: Whole blood.

## Competing interests

JRC received honoraria for lecturing from Biogen-Idec. DH has received funding for travel from Biogen Idec, Merck-Serono and Sanofi-Aventis; and received speaker honoraria from Sanofi-Aventis and Biogen Idec. LB, MK and HBS report no disclosures. PSS has served on scientific advisory boards for Biogen Idec, Merck Serono, Novartis, Genmab, TEVA, Elan and GSK; and has been on steering committees or independent data monitoring boards in clinical trials sponsored by Merck Serono, Genmab, TEVA, GSK and Bayer Schering. He has received funding of travel for these activities, has served as Editor-in-Chief of the *European Journal of Neurology*, and is an editorial board member for Therapeutic Advances in Neurological Disorders and Multiple Sclerosis.He has received speaker honoraria from Biogen Idec, Merck Serono, TEVA, Bayer Schering, Sanofi-aventis, and Novartis. His department has received research support from Biogen Idec, Bayer Schering, Merck Serono, TEVA, Baxter, Sanofi-Aventis, BioMS, Novartis, Bayer, RoFAR, Roche, Genzyme, and from the Danish Multiple Sclerosis Society, the Danish Medical Research Council, and the European Union Sixth Framework Programme: Life Sciences, Genomics and Biotechnology for Health. FS has served on scientific advisory boards for and received funding for travel from Biogen Idec, Merck-Serono, Novartis, Sanofi-Aventis and Teva; and has served as a consultant for Biogen Idec and Novo Nordisk; received speaker honoraria from Bayer-Schering, Biogen Idec, Merck-Serono, Novartis, Sanofi-Aventis and Schering-Ploug. He has received research support from Biogen Idec, Merck-Serono, Novartis and Sanofi-Aventis; and serves as section editor on *Multiple Sclerosis and Related Disorders*.

## Authors’ contributions

JRC, LB, DH, MK, HBS and FS collected the samples, performed the laboratory analyses and analyzed data. JRC and FS wrote the first draft of the manuscript. All authors participated in the interpretation of the data and contributed to the critical review of the manuscript. All authors read and approved the final version of the manuscript.
